# Editor’s introduction: The function of emotion in qualitative healthcare research

**DOI:** 10.1016/j.qrmh.2026.100035

**Published:** 2026-01-27

**Authors:** Warren Bareiss

**Affiliations:** College of Arts, Humanities, and Social Sciences, University of South Carolina Upstate, Spartanburg, USA

Arguably, one of the most salient distinctions between qualitative and quantitative research is the role of emotion. Generally, emotion is kept at a distance in quantitative methods. Researchers might measure emotional presence and effect, and, indeed, emotion might be the central factor examined. But while quantitative methodologies excel in measuring emotion from a distance, emotion is at the core of what we do in qualitative research.

I have written in a previous introduction that the primary advice I give to authors when revising their work is to tell a good story. Where is the conflict, the struggle, and the humanity in the stories we tell? Rather than quantify what participants tell us, I advise authors to let the participants speak, to tell their stories. Stories participants tell us are about the *experience* of illness and caregiving. Furthermore, our stories as authors consist of a carefully constructed patchwork of the stories told to us.

Stories told well help the reader to feel what it is like to be a patient or caregiver, building a sense of *communitas*, as anthropologist, Victor Turner (1974), described it. For Turner, *spontaneous communitas* is a transcendent state of connection often achieved through ritual; however, ritual is not the only means of affecting *communitas*. Festivals, social crises, and the experience of art are other instances of how individuals can transcend their daily routine into a liminal placeless place where ego dissolves into a vivid experience of collectivity. These occasions tend to elicit deep, overpowering emotional responses as the hard shell of the ego dissolves into a wondrous sense of possibility.

Storytelling works as a means of *communitas* by fostering empathy in multiple directions: between storytellers and listeners as well as between listeners and imagined characters. Empathy, in turn, is connection formed through shared emotions. Every article in this issue exemplifies this process.

For Eva C. van Reenen and her colleagues, the operative word is “uncertainty”—in this case, uncertainty regarding patients with relapsing-remitting multiple sclerosis (RRMS). While uncertainty, per se, is not an emotion, it is what researchers elsewhere have termed an “elicitor” and “modulator” of emotional states (Morris et al., 2022). Through their interviews with 15 patients, van Reenen et al. trace uncertainty to anxieties associated with medical spaces, technologies, patient-provider expectations, and unclear communication. Further confounding the emotional effects of uncertainty for patients—and, by extension, providers—is the largely implicit and unspoken factor in the patient-provider relationship.

Molly Harrod and her co-authors shift attention to the emotional experiences of healthcare professionals, specifically burnout among mental health providers in US Veteran Health Administration (VA) medical centers. Harrod, et al. document the emotional toll of burnout among participants, including emotional disengagement in their work and emotional exhaustion in a profession that demands empathy and connection. Analyzing interviews among an unusually large sample of 54 mental health providers in the VA system, Harrod et al. illustrate professional dilemmas that burnout conditions, notably distress about performing caregiving duties when feeling overwhelmed and even going so far as to question their trust in the medical system within which the burnout occurs.

Rebekah Cole shifts attention in yet a different direction by examining the impact of emotion among healthcare researchers in their roles as witnesses to the suffering of others. Cole takes an autoethnographic approach as she reflects upon her unpreparedness for the emotional toll that she experienced as a healthcare researcher, notably when interviewing active combatants in the Ukrainian military. Cole vividly makes the case that healthcare researchers—particularly those using qualitative methods through which they immerse themselves into the experiences of others—need and deserve better training to withstand and make use of the emotional burden of their work. Indeed, Cole goes so far as to argue that “emotional labor” required of qualitative researchers is an advantage rather than a hindrance, but without adequate training, it can lead to burnout, much as Harrod et al. describe in their contribution.

The emotional toll of recovering from stroke is peppered throughout the contribution authored by Ibrahim et al. Regular readers of *QRMH* might notice that this is the second article that we have by published by Ibrahim and her colleagues on young and middle-aged women who had experienced strokes. Whereas the previous feature focused on developing habits to facilitate brain health post-stroke, this article explores more general lifestyle factors contributing to recovery. Among questions asked participants in this study, Ibrahim specifically inquired about the role that emotions play in the adoption of healthy habits. Perhaps, not surprisingly, responses revealed a web of varying emotions accompanying recovery, further complicated and conditioned by gender roles and expectations.

As we can see already from this brief introduction, emotion is central and fundamental to each article in this issue. That is important, but not the main point that I want to emphasize. Each of these articles and the issue overall provides evidence for my assertion that qualitative research methods are particularly effective in getting at the human experience of health, illness, and caregiving, and they do so by their ability to appreciate and explore emotion in all its messy richness. As Cole argues in her feature, emotion is an asset in what we do.
